# Laparoscopic cholecystectomy for double gallbladder malformation with cystic duct variation: a case report

**DOI:** 10.3389/fsurg.2026.1899269

**Published:** 2026-07-16

**Authors:** Shaohua Yang, Xiaohui Gu, Liping Ma, Shibo Zhang, Jianwei Tang, Zhuoyu Zhao, Bo Liang, Meixiang Liang, Yumei Jin, Yongping Xu

**Affiliations:** 1 Hepatobiliary and Pancreatic Surgery Department, Qujing Central Hospital, Yunnan Province, Qujing, Yunnan, China; 2 General Medicine Department, Second People’s Hospital, Qujing, Yunnan, China; 3 Magnetic Resonance Imaging Room, Xuanwei Traditional Chinese Medicine Hospital, Xuanwei, Yunnan, China; 4 Department of Medical Imaging Center, Qujing Central Hospital, Yunnan Province, Qujing, Yunnan, China

**Keywords:** cystic duct variation, double gallbladder, gallbladder malformation, intraoperative cholangiography, laparoscopic cholecystectomy

## Abstract

**Background:**

Double gallbladder associated with cystic duct variation is a rare congenital biliary anomaly that carries a high risk of intraoperative misdiagnosis.

**Case presentation:**

We report a case involving a 20-year-old female who presented with right upper quadrant pain. Based on clinical findings and preoperative abdominal magnetic resonance imaging (MRI) with magnetic resonance cholangiopancreatography (MRCP), she was diagnosed with gallbladder stones complicated by acute cholecystitis and a double gallbladder malformation, initially classified as the Y-type variant of Harlaftis Type I. Intraoperative exploration revealed two separate gallbladders sharing a single cystic artery; however, the actual anatomy did not fully correspond to the preoperative imaging assessment. Given the presence of anomalous biliary structures and intraoperative bile leakage, an intraoperative cholangiogram (IOC) was performed to delineate the biliary anatomy. IOC showed that each gallbladder drained via its own cystic duct into the common hepatic duct: one cystic duct coursed parallel to the common hepatic duct and joined it at a low insertion point, while the other connected in the typical fashion. Integrating operative findings with IOC results, the malformation was reclassified as an H-type double gallbladder (Harlaftis Type II) with an abnormally low cystic duct confluence. Laparoscopic double cholecystectomy was successfully completed under IOC guidance.

**Conclusions:**

This case highlights that although MRI/MRCP can aid in the preoperative detection and preliminary classification of double gallbladder anomalies, imaging may not accurately reflect the true intraoperative anatomy when complex cystic duct variations are present. When intraoperative observations deviate from preoperative expectations or pose a risk of biliary injury, prompt use of IOC is essential to clarify biliary anatomy, correct diagnostic discrepancies, prevent iatrogenic injury, and ensure surgical safety.

## Introduction

Double gallbladder is a rare congenital biliary malformation with an estimated incidence of approximately 1 in 4,000 (1). Its clinical relevance extends beyond the anatomical anomaly itself, as it is frequently accompanied by variations in the cystic duct, biliary tree, and even vascular anatomy, factors that significantly complicate anatomical identification and heighten the risk of biliary injury during laparoscopic cholecystectomy (LC) (2, 3). When coexisting with common biliary pathologies such as cholelithiasis or cholecystitis, it often necessitates surgical intervention. For symptomatic patients, LC remains the treatment of choice; however, in the presence of complex anatomical variants, accurate delineation of biliary structures and prevention of iatrogenic injury are critical to safe surgical execution.

Preoperative imaging, particularly magnetic resonance cholangiopancreatography (MRCP), plays a valuable role in detecting double gallbladder malformations and enabling preliminary classification, thereby supporting preoperative risk stratification and surgical planning. Nevertheless, when complex cystic duct variations are present, the classification based on preoperative imaging may not fully correspond to the actual intraoperative anatomy. In other words, although MRCP provides essential preoperative information, it cannot replace real-time intraoperative anatomical verification. In cases featuring intricate anatomy, discrepancies between preoperative imaging and intraoperative findings, or an elevated risk of biliary injury, timely intraoperative cholangiography (IOC) to clarify the biliary anatomy (4, 5) is crucial for correcting intraoperative misjudgments, guiding subsequent steps, and preventing further complications.

Reports of double gallbladder malformations associated with complex cystic duct variations remain scarce, and cases in which preoperative imaging-based classification diverges from the intraoperative reality carry particular clinical significance. The value of such cases lies not only in illustrating a rare anomaly but also in underscoring that even thorough preoperative imaging should not lead to complacency regarding potential anatomical aberrations during surgery. Surgeons must maintain a high index of suspicion and, when indicated, employ IOC or other adjuncts to reconstruct the biliary anatomy in accordance with the principles of safe cholecystectomy. Accordingly, we report a case of a young female patient with double gallbladder and cystic duct variation. Preoperative MRCP initially suggested a Harlaftis type I Y-variant; however, intraoperative findings inconsistent with this assessment prompted IOC, which revealed a more complex configuration, specifically, a double gallbladder with abnormally low confluence of the cystic ducts. This report emphasizes not merely the rarity of the malformation but also the potential for preoperative misclassification in the setting of complex biliary anatomy, the necessity of intraoperative re-evaluation, and the pivotal role of IOC in averting biliary injury and ensuring the safety of laparoscopic surgery.

## Case presentation

A 20-year-old female patient was admitted with a chief complaint of “right upper quadrant pain persisting for 3 days.” The pain was dull in character and accompanied by intermittent burning epigastric discomfort. There was no radiation of pain; however, she reported nausea and vomiting. The vomitus consisted only of gastric contents, was non-projectile, and contained no blood. She denied diarrhea, hematochezia, chest tightness, dyspnea, palpitations, or shortness of breath. On admission physical examination, the abdomen was flat and symmetrical, with normal abdominal respiratory movements. Epigastric tenderness was present, but rebound tenderness was absent. The gallbladder was not palpable, and Murphy's sign was negative. Abdominal percussion yielded resonance throughout, with no hepatic percussion tenderness. Bowel sounds were normal at approximately 3 per minute, and no vascular murmurs were auscultated. Laboratory findings showed a white blood cell count of 19.5 × 10^9^/L, neutrophil percentage of 77.4%, C-reactive protein level of 122.98 mg/L, procalcitonin level of 0.1 μg/L, total bilirubin of 14.2 μmol/L, alanine aminotransferase of 67 U/L, aspartate aminotransferase of 35 U/L, alkaline phosphatase of 92 U/L, and γ-glutamyltranspeptidase of 49 U/L. An upper abdominal MRI and MRCP performed at Xuanwei Traditional Chinese Medicine Hospital, Yunnan Province, revealed a duplicated gallbladder (double gallbladder) malformation. Multiple stones were present in the superior gallbladder, and a single stone was located in the neck of the inferior gallbladder (Figure 1). Both gallbladders showed signs of cholecystitis, with more pronounced inflammatory changes in the superior one. MRCP demonstrated multiple scattered, round, low-signal filling defects within the lumen of the superior gallbladder, along with wall thickening, edema, and surrounding pericholecystic fluid. A low-signal filling defect was also noted in the neck of the inferior gallbladder. The two cystic ducts joined to form a single duct that connected to the extrahepatic bile duct. This common cystic duct coursed parallel to the common hepatic duct, with its confluence situated slightly inferiorly. No significant dilation was observed in the intrahepatic or extrahepatic bile ducts, common bile duct, or pancreatic duct, and no abnormal intraluminal signals were detected. Based on these imaging findings, the duplicated gallbladder was preoperatively classified as a Y-type variant of Harlaftis type I (Figure 1B). The preoperative diagnosis was cholelithiasis with acute cholecystitis and duplicated gallbladder malformation.

**Figure 1:**
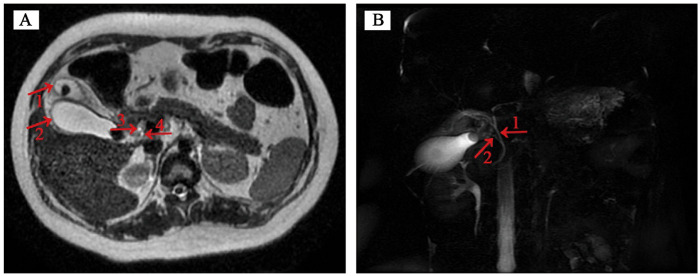
Preoperative abdominal MRI. **(A)** Two gallbladders are visible (arrows 1 and 2). Arrow 3 indicates the cystic duct; arrow 4 indicates the common hepatic duct. **(B)** MRCP shows that the two cystic ducts join to form a single cystic duct, which then drains into the common hepatic duct at a slightly inferior confluence. Arrow 1 denotes the common hepatic duct; arrow 2 denotes the cystic duct.

Emergency laparoscopic cholecystectomy was performed. Laparoscopic exploration revealed a small amount of pale yellow exudate surrounding the liver and gallbladder, with the gallbladder partially enveloped by the greater omentum. After adhesiolysis, two distinct gallbladders were identified: Gallbladder 1 was tense, markedly congested and edematous, measuring approximately 8 cm × 4 cm × 1 cm, with thickened walls and abundant pericholecystic exudate; Gallbladder 2 was less tense, showed mild congestion and edema, and measured approximately 10 cm × 4 cm × 1 cm (Figure 2A). The Calot's triangle remained well-defined, though associated lymph nodes were enlarged. The gallbladder neck was retracted with spring forceps to fully expose the Calot's triangle, and the overlying serosa was incised using an electrocautery hook. Meticulous dissection of the anterior and posterior aspects of the gallbladder triangle and the hepatoduodenal ligament was carried out to clearly delineate the cystic duct, common hepatic duct, and common bile duct. The cystic duct measured approximately 0.7 cm in length and 0.4 cm in diameter. Two bioabsorbable clips were applied to its proximal and distal segments, and the cystic duct (labeled as cystic duct 1) was then slowly transected with scissors (Figures 2B,C), yielding a small amount of bile upon division. Further dissection identified the cystic artery, which was secured with a single bioabsorbable clip and transected with scissors, exposing the vascular stump (Figures 2D–F). Subsequent dissection within the Calot's triangle revealed a duct-like structure resembling a second cystic artery (Figure 2F). This structure was clipped with a bioabsorbable clip (Figure 2G), and transection with scissors produced yellow bile, confirming it as an accessory bile duct and indicating an intraoperative biliary injury. Given the discrepancy between these intraoperative findings and the preoperative magnetic resonance imaging (MRI) results, an infusion extension tube was inserted into the injured duct, and iohexol was injected for intraoperative cholangiography (Figure 2H). The left and right hepatic ducts, common hepatic duct, common bile duct, and two cystic ducts were visualized. The first cystic duct was identified as cystic duct 1 during intraoperative dissection (Figures 2B,C and 3A), while the second cystic duct, defined as cystic duct 2, was delineated via intraoperative catheterization and cholangiography (Figures 2H, 3A). Cystic duct 1 exhibited anatomic variation, running parallel to the common hepatic duct and joining it at a low confluence; cystic duct 2 connected to the common hepatic duct in the typical configuration (Figure 3A). Intraoperative findings were consistent with a Harlaftis type II duplication featuring an H-type variant, accompanied by cystic duct anomaly. Intraoperative cholangiography demonstrated no contrast extravasation from the intrahepatic or extrahepatic bile ducts, and cystic duct 2 was secured with bioabsorbable clips (Figure 2I). Meticulous dissection identified only a single cystic artery. Postoperative examination of the resected specimen confirmed the absence of communication between the two gallbladders, with gallstones present in both (Figure 3B). Laboratory reassessment at 24 h postoperatively yielded the following results: white blood cell count 12.9 × 10^9^/L, neutrophil percentage 70.4%, C-reactive protein 121.91 mg/L, procalcitonin 0.114 μg/L, total bilirubin 11.7 μmol/L, alanine aminotransferase 44 U/L, aspartate aminotransferase 27 U/L, alkaline phosphatase 77 U/L, and γ-glutamyl transferase 38 U/L. A small omental foramen drain was placed postoperatively and monitored continuously for 48 h. Approximately 20 mL of pale yellow inflammatory exudate was collected, with no evidence of bile leakage or hemorrhage. The drain was removed at 48 h postoperatively. Pathological examination revealed two separate gallbladder cavities. In Gallbladder 1, the gallbladder contents had been lost, and it measured 7.5 cm × 4.3 cm × 2 cm in size, containing yellow, hardened stones. The gallbladder wall was rough, with a thickness of 0.6 cm. Pathological diagnosis: gallstones with acute cholecystitis and localized suppuration. Gallbladder 2 measured 4.7 cm × 1.2 cm × 0.7 cm, with its contents also lost. The gallbladder wall was 0.1 cm thick and contained a brown stone approximately 1.2 cm in diameter. Pathological diagnosis: gallstones with chronic cholecystitis. One month postoperatively, a non-contrast MRI and MRCP of the upper abdomen were performed at Qujing Central Hospital, Yunnan Province. Findings showed a few patchy T2 fat-suppressed hyperintense signals in the surgical bed, with no perihilar fluid collection. On coronal imaging, the cystic duct and common hepatic duct formed an inverted “8” configuration (Figure 3C). MRCP confirmed that the cystic duct ran parallel to the common hepatic duct and joined it at a low position (Figure 3D). No focal stenosis or dilation was observed in the common hepatic or common bile ducts, and no intraluminal filling defects were detected.

**Figure 2:**
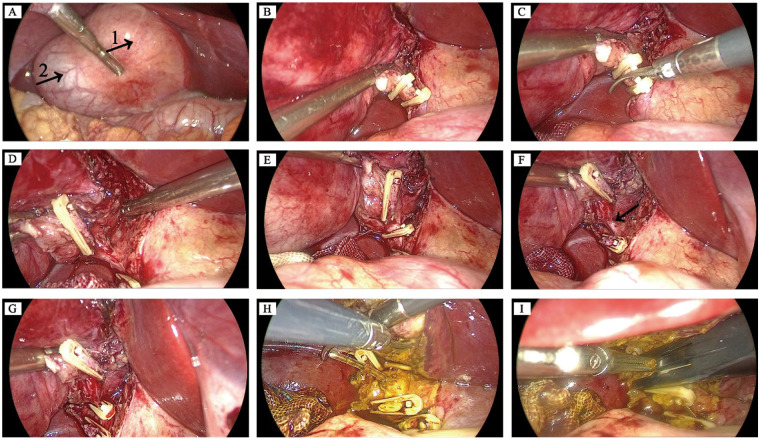
Intraoperative findings. **(A)** Two gallbladders were identified, each showing differing degrees of inflammatory edema. **(B)** The cystic duct (cystic duct 1) was occluded with bioabsorbable clips. **(C)** The cystic duct (cystic duct 1) was transected using surgical scissors. **(D)** The cystic artery was visualized. **(E)** The cystic artery was secured with a bioabsorbable clip. **(F)** After transection of the cystic artery, a duct entering the gallbladder was noted (black arrow), initially presumed to be a second cystic artery. **(G)** This duct was clipped with a bioabsorbable clip. **(H)** Incision with scissors released yellow bile, confirming it as a bile duct; intraoperative cholangiography was then performed. **(I)** Following cholangiographic confirmation that the structure was cystic duct (cystic duct 2), it was occluded with bioabsorbable clips.

**Figure 3:**
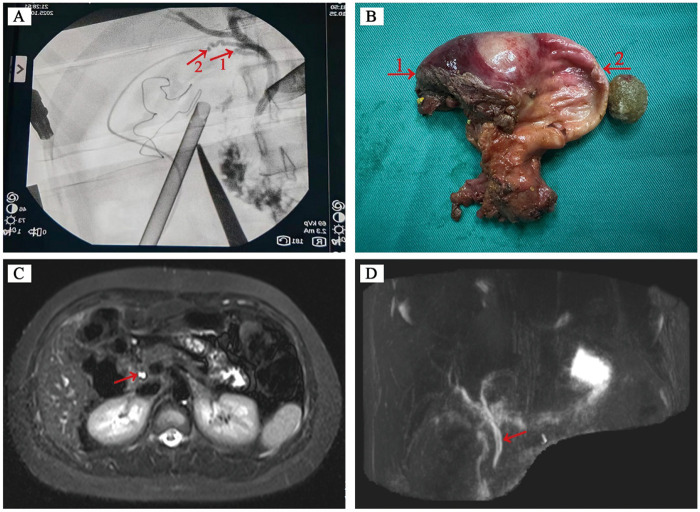
**(A)** intraoperative cholangiography identified cystic duct 1 (red arrow 1) and cystic duct 2 (red arrow 2). Cystic duct 1 showed anatomic variation, joining the common hepatic duct at a low confluence, whereas cystic duct 2 connected to the common hepatic duct in the typical configuration. **(B)** Postoperative gallbladder specimens contained gallstones in both gallbladders. Gallbladder 1 (red arrow 1) exhibited features of acute inflammation, while gallbladder 2 (red arrow 2) showed chronic inflammatory changes. **(C)** Coronal MR imaging revealed an inverted “8” configuration between the cystic duct and the common hepatic duct (red arrow). **(D)** MRCP demonstrated the low-position confluence of the cystic duct and the common hepatic duct (red arrow).

## Discussion and conclusions

Double gallbladder is a rare congenital biliary malformation that arises from abnormal division of a single gallbladder during the fifth to sixth week of embryonic development (6). Although its overall incidence is low, its clinical importance stems from its frequent association with anatomical variations in the cystic duct, biliary tract, and even vasculature, which substantially increases the difficulty of intraoperative identification and the risk of iatrogenic injury during laparoscopic cholecystectomy (1, 2). Unlike typical cases of gallstone disease or cholecystitis, the primary challenge posed by double gallbladder is not whether resection is indicated, but rather how to perform a safe resection in the presence of complex anatomical variations while avoiding complications such as bile duct injury, bile leakage, vascular damage, and residual gallbladder tissue (7).

Classification of double gallbladder malformations typically follows the Boyden or Harlaftis systems (1, 8). The Boyden classification is based on the number of cystic ducts and the site of their confluence (9), whereas the Harlaftis classification reflects embryologic origin (10, 11) and is more widely used in clinical practice. It includes: Type I (a single cystic duct draining into the common bile duct, encompassing septated, V-shaped, and Y-shaped gallbladders); Type II (a main gallbladder and an accessory gallbladder each draining independently into the common hepatic duct via separate cystic ducts); and Type III (anomalies that do not fit Types I or II, such as triple gallbladders or other rare variants). Among these, Type II is the most common form in the Harlaftis classification (12), accounting for approximately 50% of cases (13). In this case, preoperative MRCP suggested a Y-shaped variant consistent with Harlaftis Type I; however, intraoperative cholangiography revealed a malformation resembling an H-shaped variant of Harlaftis Type II, distinguished by the presence of cystic duct variation and a low confluence of the cystic duct with the common hepatic duct (Figure 3A). The discrepancy between preoperative MRCP and intraoperative findings may be attributed to overlapping imaging planes, suboptimal scan orientation relative to the long axis of the cystic duct, artifacts from bowel gas or respiratory motion, or mucosal edema of the cystic duct secondary to acute cholecystitis. Failure to correctly identify double gallbladder and associated cystic duct anomalies during surgery can result in residual gallbladder tissue, bile duct injury, bile leakage, or biliary stricture (11, 14). Given the complexity and rarity of these anatomical configurations, preoperative imaging, particularly ultrasound, often fails to establish an accurate diagnosis (1). MRCP or contrast-enhanced abdominal CT provides clearer delineation of biliary anatomy, which is essential for definitive preoperative diagnosis, assessment of cystic duct confluence patterns, and detection of potential biliary variations. These modalities are therefore critical for formulating a safe surgical strategy (7, 15). Inadequate preoperative recognition of such anatomical variants remains a leading contributor to heightened risks of bile duct injury, postoperative bile leakage, and recurrent gallstone disease (8, 16).

For symptomatic patients with double gallbladders, LC remains the treatment of choice, though a more cautious surgical approach is warranted. To prevent omission of a diseased gallbladder and the need for reoperation, the procedure must entail complete removal of both gallbladders (15). The primary intraoperative challenge lies in identifying and managing two cystic ducts and two cystic arteries within Calot's triangle, a complexity that inherently elevates the risks of hemorrhage and inadvertent injury to the common bile duct or hepatic duct. In the absence of a discernible cystic artery, the gallbladder may receive blood supply from the microvascular plexus on the “hepatic bed surface” where it contacts the liver, or via collateral circulation between the cystic duct artery and other small branches of the hepatic artery. In this case, only one cystic artery was identified intraoperatively. The absence of a second cystic artery increases the risk of misidentifying the right hepatic artery as the cystic artery, potentially leading to major vascular injury. Therefore, strict adherence to the principles of “safe cholecystectomy” is paramount (17). Surgeons should aim to achieve the “critical view of safety” (CVS) for both the cystic duct and cystic artery. When confronted with complex biliary variations, unclear anatomy, or any uncertainty, IOC is strongly recommended to delineate biliary anatomy (4), mitigate the risk of iatrogenic biliary injury (15, 17), and facilitate early recognition of such injuries, thereby improving the likelihood of successful biliary repair. Additionally, intraoperative laparoscopic ultrasound (LUS) and indocyanine green fluorescence cholangiography (ICG-FC) can further clarify biliary anatomy, reduce the risk of biliary and vascular complications, and enhance overall procedural safety (18). In this case, the safe and complete laparoscopic removal of both gallbladders was likely attributable to the surgeon's recognition of intraoperative anatomical discrepancies with preoperative imaging, heightened vigilance regarding potential variations, and timely use of IOC to confirm biliary anatomy. One month postoperatively, the patient underwent follow-up examinations including upper abdominal magnetic resonance imaging (MRI) and magnetic resonance cholangiopancreatography (MRCP). Coronal MRI images demonstrated an inverted “8” configuration between the cystic duct and common hepatic duct. MRCP further revealed that the variant cystic duct ran parallel to the common hepatic duct and converged at a low level. Notably, the elongated variant cystic duct was not completely resected, which may predispose the patient to postoperative complications such as upper abdominal pain, abdominal distension, and gallstone recurrence, thereby elevating the risk of postcholecystectomy syndrome. When managing complex scenarios such as double gallbladder, particularly when severe adhesions, inflammation, or ambiguous anatomy preclude safe exposure of Calot's triangle, the surgeon must be prepared to implement alternative strategies. Subtotal cholecystectomy, for instance, represents an effective option to avoid catastrophic biliary injury (19, 20). Furthermore, employing the “bottom-up, top-down, retrograde approach” may also aid in completing the procedure under challenging conditions (19). The successful outcome in this case underscores that meticulous preoperative planning combined with adaptable intraoperative decision-making is essential for ensuring surgical safety and preventing serious complications in rare biliary anatomical variants.

Although double gallbladder malformation complicated by cystic duct variations is rare, its clinical risks arise not from the malformation itself but from the associated complex biliary anatomy, which can lead to inaccurate preoperative classification and intraoperative misinterpretation of anatomical structures. This case demonstrates that MRI/MRCP aids in the preoperative detection of double gallbladder malformation and provides a preliminary anatomical assessment. However, when complex cystic duct anomalies are present, preoperative imaging findings cannot replace dynamic intraoperative confirmation of anatomy. If intraoperative observations conflict with preoperative imaging or if there is concern for potential biliary injury, timely use of IOC to delineate the biliary tract is essential for correcting intraoperative misjudgments, preventing further biliary damage, and ensuring the safe completion of laparoscopic surgery. In managing such rare and high-risk biliary variants, meticulous preoperative planning, heightened awareness of atypical anatomy, and adaptable intraoperative decision-making carry greater clinical value than the mere achievement of successful resection.

## Data Availability

The original contributions presented in the study are included in the article/Supplementary Material, further inquiries can be directed to the corresponding author.
